# Skin Diseases and Syphilis

**Published:** 1894-04

**Authors:** 


### SKIN DISEASES AND SYPHILIS.

EDITED BY DR. M. B. HUTCHINS.

### VACCINATION—DISEASES.

Epstine (*Jahrbuck für Kinderheilkunde,* 1893, 35, *p.* 442). (1) Two cases of hemorrhagic condition following vaccination out of four hundred and thirty vaccinated with bovine virus. One, a somewhat rachitic child, had an erythema vaccinosum four days afterwards, as well as hemorrhagic spots on the extensor surfaces of the extremities. No blood in the vaccine blebs. On the eighth day, as the spots faded, fever, conjunctivitis and coryza. Measles eruption three days later. Infection with measles preceded vaccination, but the hemorrhages were caused by the latter.

The second child had fever on the sixth day, and on the eighth numerous hemorrhages (intra-dermal) on the body and extremities. These were resorbed in six days. No blood in vaccination lesions.

(2) Among four hundred and thirty vaccinated persons there were fourteen cases of vaccinal erythema. The eruption occurred not earlier than the fourth, usually on the seventh, day, simultaneous with the acme of the vaccination symptoms. Localization variable, mostly around the point of inoculation, next on the extensors of the extremities, least frequently on the sides of the thorax. The eruption resembles measles, from millet seed to "dollar" size, roundish, half-moon form, irregular, map-like, bluish—to dark carmine—red but brighter than the measles eruption. Development gradually for two or three days, mostly with fever. Duration two to eight days.—*M. f. P. D., XVIII., No.* 5.

An Atlanta physician was lately vaccinated. In due course of time the local symptoms became severe. At the height of these one or two chills occurred per diem, with, at least once, a rise of temperature to 104°. Then a macular eruption of pinkish color appeared in inguina, later just above the waist, then on back (scattering), and thickly on buttocks, especially over tuberosities. Individual lesions about finger-nail size, which tended to confluence. On the back and buttocks a slight, flattish, papular elevation of the middle of the maculæ, and those of back and buttocks somewhat brighter red than the others. Itching rather marked. Began fading in four or five days with a faint desquamation. M. B. H.

---

### GANGRENE OF THE GENITALS.

Clinical lecture by Balzer. (*Med. Moderne*, 1893, *No.* 55.) He described twenty-five cases, eight of which were from paraphimosis, eight from soft chancre, and nine of unknown cause. He remarked the tolerably frequent termination of soft chancre in gangrene. They are different processes. [Some remarks on micro-organisms here.] The processes do not develop simultaneously, the ulceration precedes the gangrene, former opening the way for the latter under the influence of uncleanliness, badly fitting dressings, etc.—*M. für P. D., XVIII., No.* 1.

---

### SYPHILIS AND MARRIAGE.

L. Manning (*Giorn. ital. d. Mal. ven. e. d. pelle.*) takes into consideration the extremely momentous importance of the syphilitic infection both for families and individuals, as well as the fact that syphilis is mainly brought into the family by the man. He, therefore, desires legal acts prohibiting marriage in the active secondary stage, allowing it only after careful specific treatment for many years and the complete absence of symptoms for ten to twelve months.—*Monatshefte für Prak. Derm. XVII., No.* 12.

---

### WEAKENING OF SYPHILIS.

*M. für Prak. Derm., XVII., No.* 12, quoting *Prog. Med.* C. Paul spoke on this subject in *Société de Therapeutique*, this diminution of syphilitic action being shown in the symptoms being limited to a chancre, or some trivial manifestation. Thus appeared two infected young people whose mothers were syphilitic, without the former being congenitally diseased. Their cases were entirely simple—"rudimentary." He asks if this is not explainable by the fathers having infected the mothers.

### UNUSUAL SITES OF SYPHILITIC INFECTION.

Peter (*Berl. Klin. Wochenschr*, 1893, *No.* 30) in Lassar's polyclinic, in 1892, saw twenty-four cases in which the infection did not occur from coitus, but twice in the mouth from unnatural sexual intercourse, the rest from kissing, shaving, etc. Of especial interest is the initial lesion in children, as it may be either overlooked or confused with something else. Later, such children show "late" symptoms, and are put under the head of "late hereditary syphilis."—*M. für P. D., XVII., No.* 12.

### CURE OF A CASE OF LUPUS ERYTHEMATOSUS WITH KOCH'S TUBERCULIN INJECTION.

Sechi (*Rif. Med.*, 1893, *No.* 169) reports such a cure, and finds herein a new proof (? H.) for the tubercular nature of lupus erythematosus, and emphasizes the value of tuberculin in diagnosis.—*M. f. P. D., XVIII., No.* 1.

[Decidedly non-tubercular diseases react to tuberculin injections.—ED.]

### OPERATIONS FOR EPITHELIOMA NASI.

Goris presented in the Brussels polyclinic a sixty-year old patient with extensive epithelioma of the nose, which had been operated on several times, always followed by recurrence. Finally Goris totally removed the nose, resected the upper jaw, and made a rhinoplasty after Langenbeck. (*Presse Med. Belge*, 1893, *No.* 30.)—*M. für P. D., XVIII., No.* 1.

